# Alternate antigen processing in the presence of gamma interferon may misdirect the immune recognition of cancer

**DOI:** 10.1186/2051-1426-3-S2-P453

**Published:** 2015-11-04

**Authors:** Katherine Woods, Ralf B Schittenhelm, Dani Heesterbeek, Ashley Knights, Matthew Anaka, Anthony W Purcell, Andreas Behren, Jonathan Cebon

**Affiliations:** 1 Olivia Newton-John Cancer Research Institute, Melbourne, Australia; 2 Monash University, Melbourne, Australia; 3 Olivia Newton-John Cancer Research Institute, Heidelberg, Australia

## 

Degradation of cellular proteins by the proteasome is critical for the generation of MHC-associated peptides. The constitutive proteasome and the IFNγ-induced immunoproteasome (IP) differ in the use of three catalytic β subunits, which alters production of MHC class I epitopes. The potential for a disparate repertoire of epitopes produced between inflammatory/non-inflammatory tumours therefore arises.

We have extensively investigated this phenomenon both broadly, in terms of the cancer cell as a whole, and specifically, by evaluating presentation of three NY-ESO-1 HLA-Cw3 restricted epitopes.

We profiled the immunopeptidome of a melanoma cell line under steady state or IFNγ-treated conditions; by mass spectrometry analysis of HLA Class I bound peptides. Profound changes in the overall epitope profile presented on HLA Class I molecules, leading to extensive alteration in the overall immunogenicity of the cell, were observed.

We studied processing and presentation of three NY-ESO-1 HLA-Cw3 restricted epitopes by melanoma cell lines. Using specific T-lymphocyte clones, we examined presentation of each epitope following processing through proteasome subtypes. We further investigated processing of these epitopes by selective inhibition of the IP catalytic subunit LMP7 by siRNA knockdown, or small molecule inhibition.

*In vivo*, we demonstrate that melanoma cells can be induced to switch proteasome subtype following treatments which induce IFNγ at the tumour site.

Our data demonstrate broad changes in epitopes presented by melanoma cells under inflammatory versus non-inflammatory conditions. These results illustrate a little-studied mechanism of immune escape by tumour cells. Awareness of how individual cancer epitopes are processed by melanoma cells is therefore critical to inform development of future therapies which involve cancer vaccination, adoptive T-lymphocyte transfer, or combination treatments including these.

